# Prosthetically Guided Orthodontics (PGO): A Personalized Clinical Approach for Aesthetic Solutions Using Digital Technology

**DOI:** 10.3390/jpm12101716

**Published:** 2022-10-14

**Authors:** Pietro Venezia, Vincenzo Ronsivalle, Gaetano Isola, Ferdinando Ruiz, Emilia Casiello, Rosalia Leonardi, Antonino Lo Giudice

**Affiliations:** 1 Department of General Surgery and Surgical-Medical Specialties, School of Dentistry, University of Catania, Via S. Sofia 78, 95124 Catania, Italy; 2 Private Practice, 70121 Bari, Italy

**Keywords:** orthodontics, prosthodontics, dental aesthetics

## Abstract

Conformative rehabilitation generally involves the treatment of partial dentate or the application of veneers. In this regard, conformative rehabilitation aims to generate an aesthetic prosthetic solution minimizing the amount of tissue removal without generating occlusal input interfering with the equilibrium of neuro-muscular function. In fact, pre-prosthetic orthodontics aims to re-establishing the correct position/inclination of the neighboring or antagonist teeth, providing appropriate space for the prosthetic crown. Clear aligners therapy (CAT) represents a valuable tool in the management of prosthetic cases with a conformative approach, as it allows clinicians to plan orthodontic movements that are guided by the prosthetic outcomes. In the present manuscript, we argue the concept of prosthetic guided orthodontics (PGO) by presenting and discussing three cases treated with the Invisalign GO system, which has been developed for the clinical management of multidisciplinary orthodontic-prosthetic cases with a conformative approach. In this regard, the rationale of this paper is to address the effectiveness and predictability of the digital set-up and CAT for aesthetic conformative rehabilitations.

## 1. Introduction

Prosthetic and orthodontic treatments are often integral to multidisciplinary oral rehabilitation procedures. The combined approach aims to optimize dentofacial aesthetics and improve masticatory function, oral hygiene, and the effectiveness of the abutments [1]. Pre-prosthetic orthodontics involves moving teeth to the correct position/inclination to better support fixed prosthetic rehabilitation. In particular, pre-prosthetic orthodontics allows for an increase in the long-term prognosis of the prosthesis since the occlusal forces can be directed against the long axes of the teeth to minimize tooth preparation and tissue removal (enamel and dentin) [2]. Additionally, the interdisciplinary approach can be cost-effective for patients and clinicians, producing more stable, long-lasting, and aesthetic rehabilitation.

From a clinical perspective, multidisciplinary prosthetic treatments rely on two approaches, i.e., comprehensive rehabilitation and conformative rehabilitation. Comprehensive rehabilitation is characterized by a higher level of complexity. It involves major orthodontics movements to create space for prosthetics rehabilitation and to re-establish the occlusal plane and vertical dimension. Patients requiring comprehensive rehabilitation often need to re-establish normal occlusal and dynamic schemes besides smile aesthetics. In such cases, orthodontic therapy involving specific biomechanics and anchorage systems is required to generate complex dental movement and to eliminate dentoalveolar compensations that have occurred over time. Generally, comprehensive prosthetic rehabilitations can be listed into four categories, according to specific clinical characteristics, i.e., (1) multiple missing teeth, (2) worn anterior dentition, (3) excessive vertical overbite, and (4) tilted molars [3,4,5,6,7,8,9,10,11]. Conversely, conformative rehabilitation is characterized by minor corrections of teeth position without significant change of static or dynamic occlusion. Conformative rehabilitations generally involve the treatment of partial dentate or the application of veneers [12,13]. In this case, it is essential to reach the aesthetic outcomes and patients’ expectations, respecting the orofacial biological and functional principles. In this regard, prosthetic treatment aims to generate an aesthetic solution minimizing the amount of tissue removal and avoiding the development of occlusal interferences that could alter the equilibrium of orofacial neuro-muscular systems [14,15]. Orthodontic treatments for conformative rehabilitation generally involve correcting the position or inclination of the neighboring or antagonist teeth, providing appropriate space for the prosthetic crown, or optimizing periodontal support [16,17,18,19,20]. Furthermore, minor orthodontic corrections can be required to symmetrize the distribution of the mesiodistal and vertical prosthetic spaces. This approach allows for a harmonious and conservative preparation of the rehabilitated dentition and improves the fitting of the restoration without producing changes in the vertical dimension and occlusion. Generally, these cases are where the patients’ compliance is predominantly the improvement of smile aesthetics, and clinicians are called to perform prosthetic rehabilitations balancing oral function, oral biology, and the aesthetic outcome.

Recent advancements in digital technology have notably influenced the conventional clinical workflow by providing tools that allow dentists and orthodontists to plan multidisciplinary rehabilitative treatments more efficiently and effectively. Among digital technologies, clear aligners therapy (CAT) has represented the great revolution in modern orthodontics and is widely used as an aesthetic solution for orthodontic treatment [21]. The popularity of CAT has been fueled by the increasing demand of adult patients for a highly aesthetic appliance that straightens teeth without affecting social lives or relationships [22]. Moreover, CAT has represented a “game changer” for orthodontists since it challenges the conventional thinking of how orthodontists move teeth. In particular, CAT uses digital technology to customize the biomechanics by staging tooth movements in a specific sequence in the software program, which allows for the previsualization of the outcomes. This orthodontic system represents a valuable tool in the management of prosthetic cases with a conformative approach, as it allows clinicians to plan orthodontic movements guided by the prosthetic outcomes, optimizing the effectiveness and efficiency of aesthetic rehabilitations and respecting the biological parameters according to the conservative approach. In this manuscript, we argue the concept of prosthetic guided orthodontics (PGO) by presenting and discussing different cases treated with the clear aligner system Invisalign GO, developed for the clinical management of multidisciplinary orthodontic-prosthetic cases with a conformative approach. In this regard, the rationale of this paper is to address the effectiveness and predictability of the digital set-up and CAT for prosthetic purposes compared to previous case reports or case series generally focused on pre-prosthetic orthodontics.

### 1.1. Case 1

**Figure 1:**
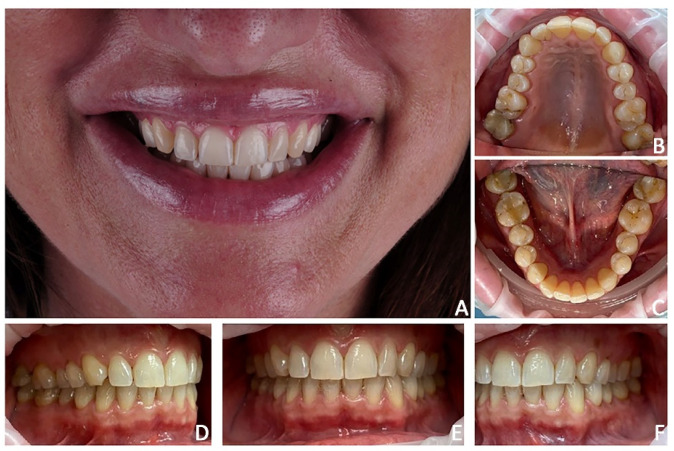
Case 1. Pre-treatment patient characteristics: (**A**) extra-oral smile view; (**B**) intra-oral upper occlusal view; (**C**) intra-oral lower occlusal view; (**D**) intra-oral lateral right view; (**E**) intra-oral frontal view; (**F**) intra-oral lateral left view.

A 48-year-old female attended a consultation to enhance smile aesthetics. Her chief complaint was the narrow upper arch with black corridors while smiling. The clinical examination revealed irregularity of the incisal margins, mild crowding, mild upper transverse contraction, particularly in the premolars region and narrowed smile with wide buccal corridors, and discrepancy of labial/dental volumes, exacerbated by previous labial filler (Figure 1).

**Figure 2:**
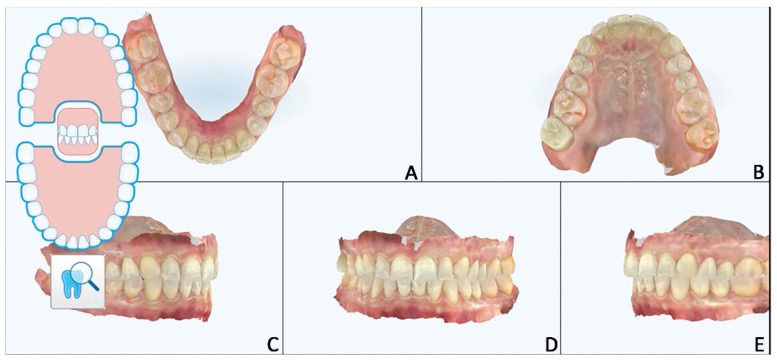
Case 1. Pre-treatment patient characteristics: intra-oral scan records. (**A**) Intra-oral lower occlusal view; (**B**) intra-oral upper occlusal view; (**C**) intra-oral lateral right view; (**D**) intra-oral frontal view; (**E**) intra-oral lateral left view.

**Figure 3:**
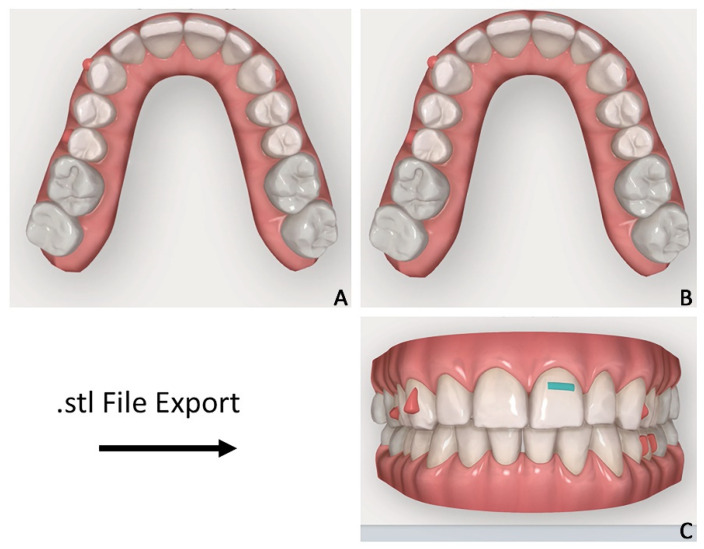
Case 1. Orthodontic set-up for staging movement for clear aligners therapy (Invisalign Go). (**A**) Pre-staged occlusal view; (**B**) post-staged occlusal view; (**C**) post-staged frontal view.

**Figure 4:**
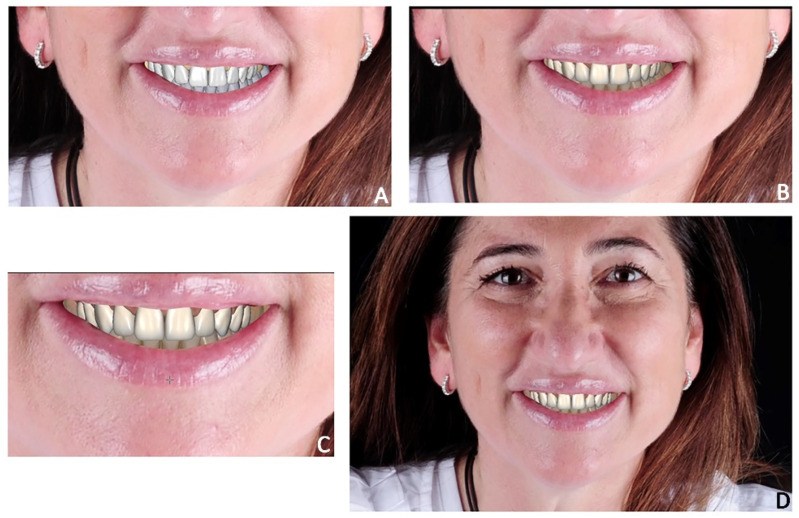
Case 1. Digital wax-up superimposed onto 2D facial photograph. (**A**,**B**) Color selection of veneers within low-third extra-oral view; (**C**) closer smile view; (**D**) facial extra-oral view.

**Figure 5:**
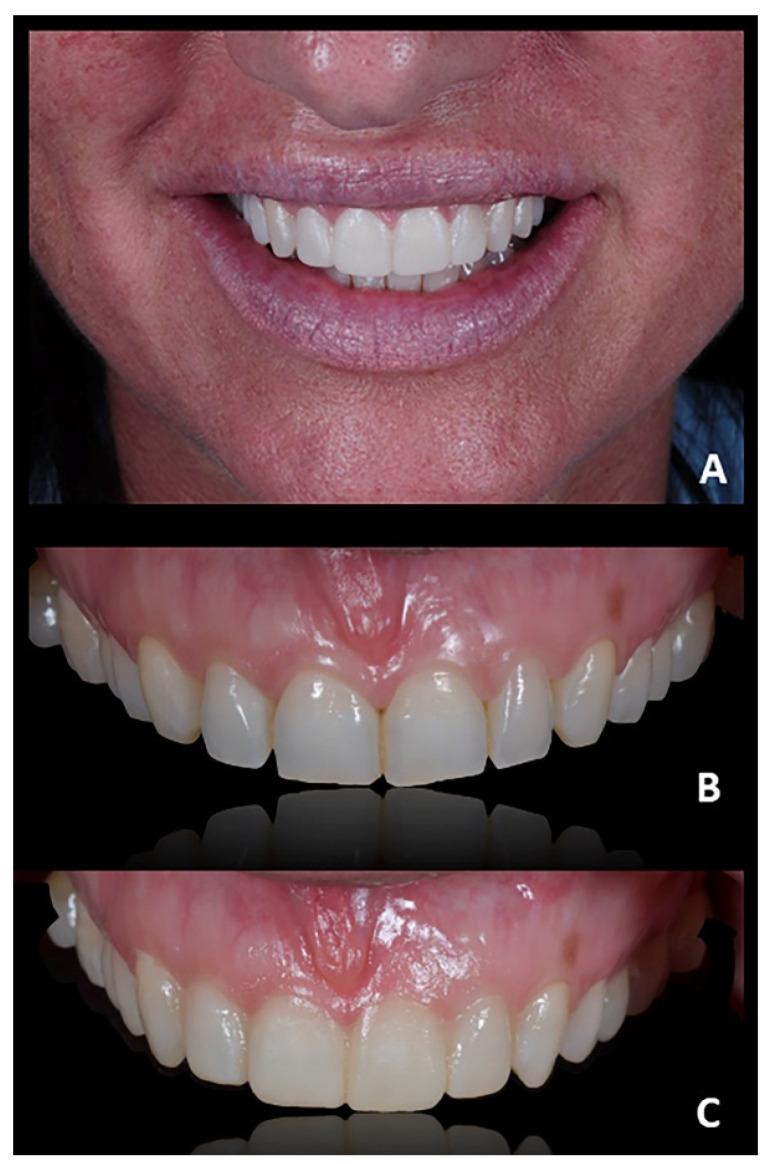
Case 1. (**A**) Pre-treatment composite mock-up; (**B**) dental alignment after orthodontic treatment; (**C**) final mock-up.

The digital diagnostic workflow, treatment strategy, and execution were performed as follows:

1. Intra-oral scanning and photo shooting (Figure 2)

2. Orthodontic set-up. The Clincheck software (Invisalign GO) was used for evaluating dental occlusion and planning pre-prosthetic tooth movements (Figure 3). Afterward, the file of the final dental position was exported from the Invisalign GO digital platform. This file allows the dental technician to optimize the design of the veneers with reference to the final dental positions.

3. Virtual waxing. The .stl file was imported into Exocad software (Align Technology, Tempe, AZ, USA), and the virtual wax-up was performed according to the desired teeth position. The final project included 10 veneers from the upper right second premolar to the upper left second premolar and was superimposed on the two-dimensional photos of the patient to evaluate the consistency in relation to the aesthetic parameters of the face (Figure 4).

4. Generation of the mock-up and validation of the project. According to the digital project, the diagnostic wax-up was prototyped, and the silicon key for the intra-oral mock-up was generated (Figure 5A). The authors recommend using a rigid laboratory silicone (platinum 85—Zhermack). The silicon key was filled with a self-hardening composite material with high aesthetic performance; in this case, we used the composite Acrytemp A2 (Zhermack). The mock-up clinical test represents a critical stage in the rehabilitation process since the mock-up enhances the communication with the patient showing a realistic preview of the final aesthetic rehabilitation and providing clinicians with a better understanding of the patient’s aesthetic expectations [9,10]. As a consequence, the patient’s satisfaction with the treatment strictly depends on the consistency of the final product with the mock-up [11,12]. Finally, this stage is important to clinically test and validate the functional adaptability of the prosthetic project.
5. Orthodontic treatment. Orthodontic movements were programmed according to the final prosthetic rehabilitations, following the principle of PGO. In this regard, the Invisalign GO system achieved predictable results with the maximum satisfaction of both the patient and the doctor (Figure 5B). The programmed orthodontic movement was staged, and eight aligners were required to achieve the final pre-prosthetic teeth position. Each aligner was worn for two weeks for a total treatment time of 4 months.

After orthodontic treatment, an intra-oral scan was performed to register the final position of the teeth. A new silicon key was produced to apply the final mock-up, which would serve as a customized guide to set the depth of the tooth preparation for the application of veneers (Figure 5C). Additionally, the authors found that the validation mock-up (i.e., the mock-up generated from the orthodontic set-up and used for the pre-visualization of the outcomes) fit perfectly after the orthodontic treatment, confirming that the programmed orthodontic movement with CAT was predictable, especially for minor corrections as those required in the prosthetic conformative approach. In light of this clinical experience, it could be postulated that clinicians may even use the pre-visualization silicone key for generating the final mock-up; however, in the absence of solid scientific evidence, the authors suggest evaluating this approach on a single-case base.

**Figure 6:**
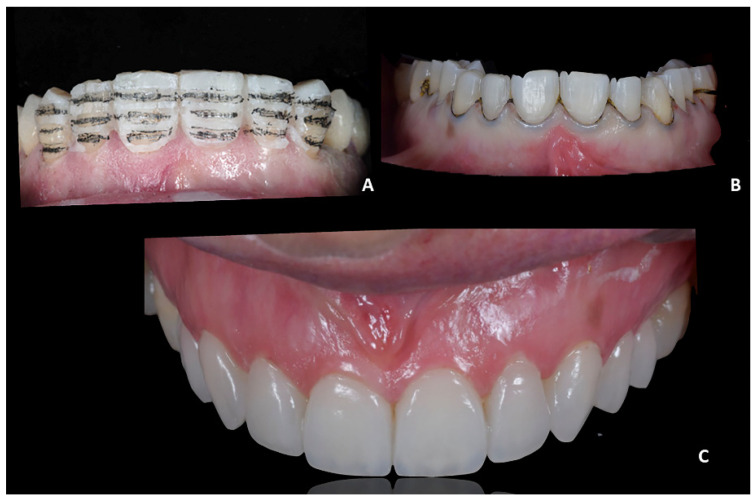
Case 1. (**A**) The mock-up served as reference guide to define the thicknesses of the prosthetic preparation; (**B**) teeth preparation; (**C**) final ceramic veneers and aesthetic rehabilitation.

**Figure 7:**
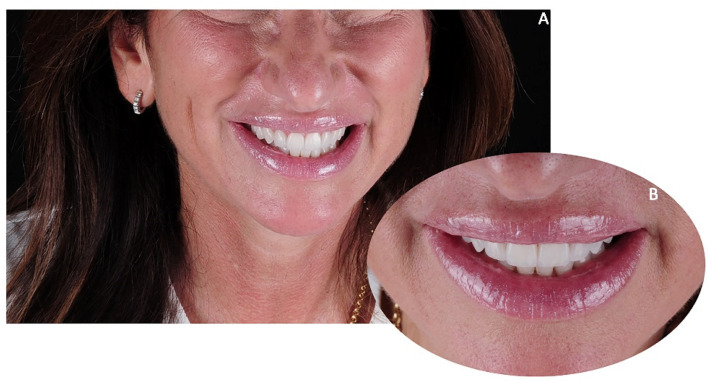
Case 1. Post-treatment extra-oral frontal view. (**A**) frontal view; (**B**) smile view.

6. Preparation technique for veneers. The prosthetic preparation was performed through the mock-up, i.e., the mock-up served as a reference guide to defining the thicknesses of the prosthetic preparation (Figure 6A). For this purpose, calibrated drills provided great predictability during tissue/material removal maneuvers. Using the mock-up as a preparation guide and following the additive design of the veneers, the preparation was maintained within the biological enamel thicknesses, minimizing the invasiveness of the procedure and allowing optimal adhesion of the ceramic veneers to the dental substrate (Figure 6B) [23]. Afterward, according to the digital project, an intra-oral scan was registered and sent to the laboratory for the finalization of the felspathic porcelain veneers.
7. Cementation of the veneers. The first step consisted of testing each veneer individually to assess the accuracy of the margins; afterward, the veneers were tested together to analyze the extent and accuracy of the contact points. Subsequently, tests with glycerine pastes (Variolink Esthetic try-in paste) were performed to choose the appropriate brightness value of the cement. Finally, the cementation of the veneers was performed according to the conventional cementation protocols, i.e., etching the ceramic with hydrochloric acid and using an aesthetic light and dual-cure luting composite for the permanent seating of veneers (Variolink Esthetic LC—Ivoclar) (Figure 6C). The aesthetic result fully satisfied both the patient and the authors and reflected the planned outcomes according to digital flow (Figure 7).

### 1.2. Case 2

**Figure 8:**
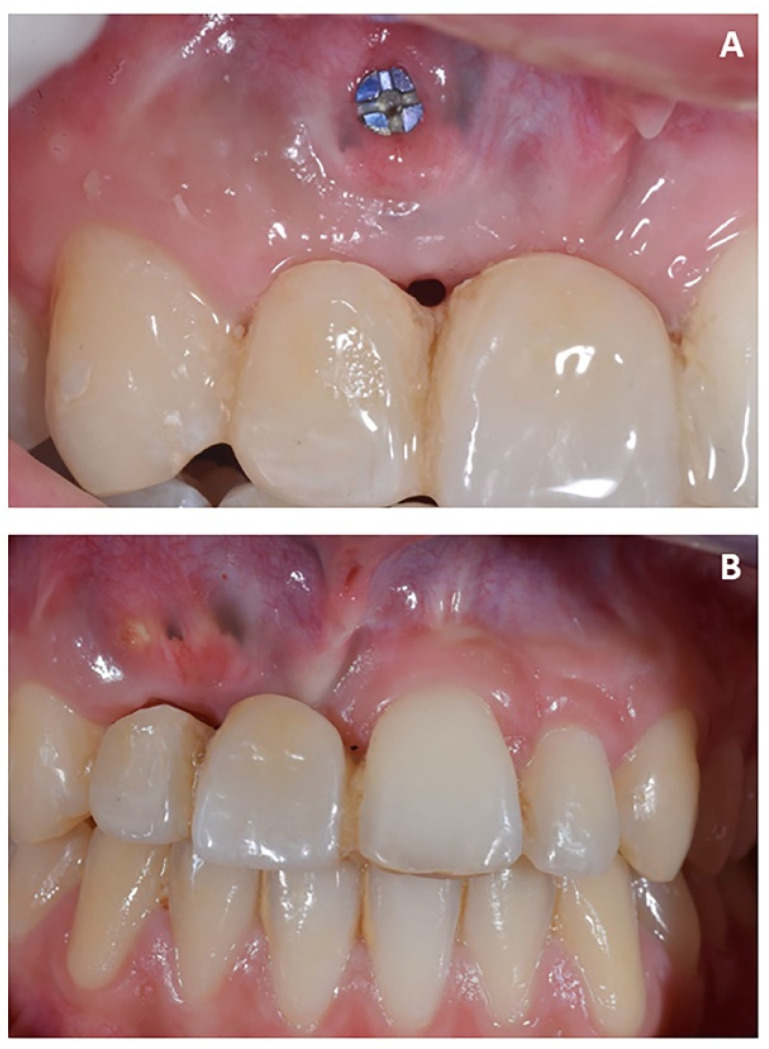
Case 2. Pre-treatment condition. (**A**) Maryland with fiberglass and resin teeth, repaired several times and interfering with proper maneuvers of oral hygiene; (**B**) absence of soft-tissue inflammation after oral hygiene and scaling.

A 24-year-old female attended a consultation for prosthetic treatment after several treatment failures. Her chief complaint was to enhance the smile’s aesthetic and possibly receive a fixed prosthesis without involving natural teeth. The patient previously underwent endodontic treatment of 1.1 and 1.2 after a traumatic injury in childhood. Subsequently, the same elements were treated with endodontic surgery and extracted after repeated abscess episodes. The last treatment, performed in adulthood, consisted of bone reconstruction by using a bone block graft to provide structural support to the anterior segment and for implant placement; however, even this solution failed, as confirmed by the exposure of the fixing screw and the inflamed surrounding tissues. The patient wore a Maryland with fiberglass and resin teeth that was repaired several times and interfered with proper maneuvers of oral hygiene (Figure 8A).

**Figure 9:**
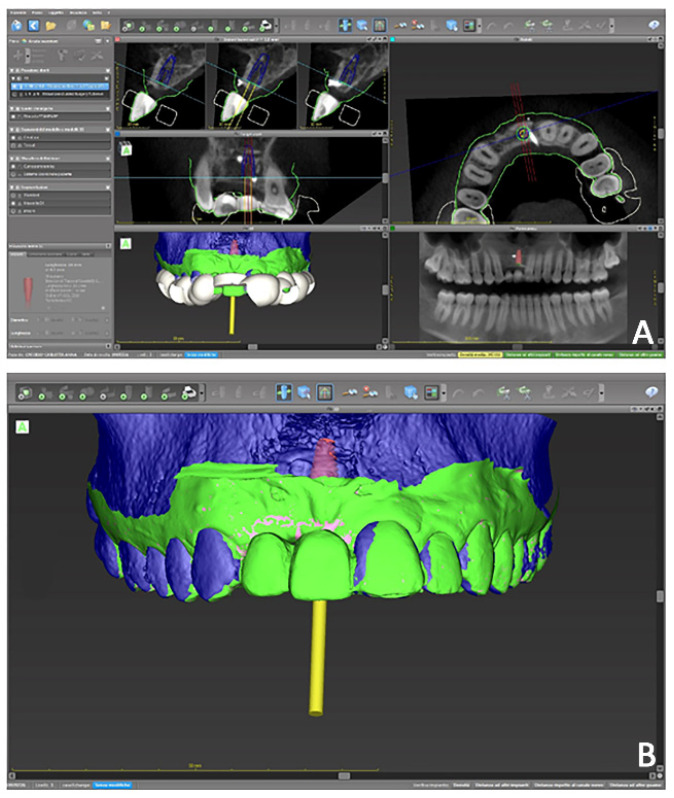
Case 2. Digital planning of implant placement and design of surgery guide. (**A**) Multiple 2D and 3D view; (**B**) 3D prosthetically guided implant surgery simulation.

**Figure 10:**
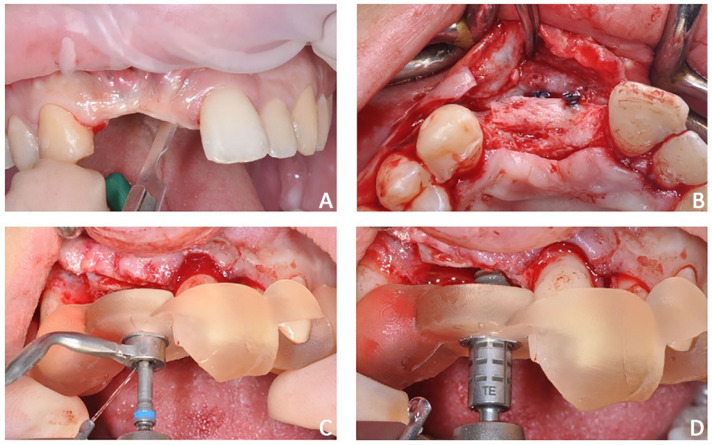
Case 2. Clinical procedure of the guided implant surgery. (**A**) flap incision, (**B**) flap elevation, (**C**) placement of surgical guide, and (**D**) guided implant placement.

**Figure 11:**
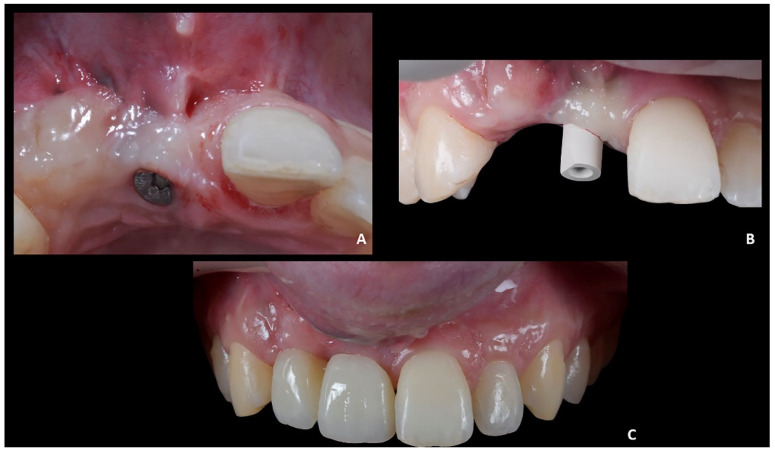
Case 2. (**A**) Healing abutment; (**B**) intra-oral scan with scan-body to detect the axial orientation of the implant; (**C**) prosthetic restoration.

**Figure 12:**
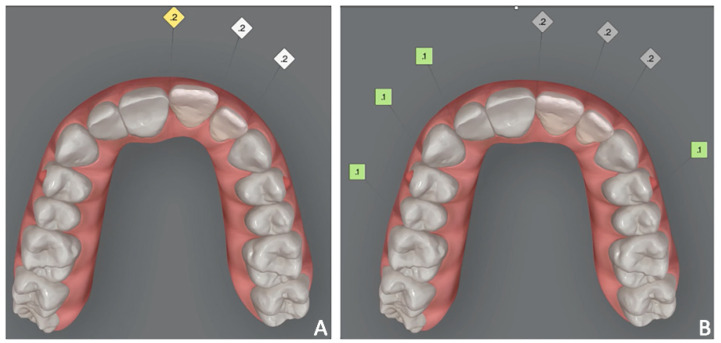
Case 2. Orthodontic set-up. (**A**) pre-treatment; (**B**) simulated final position of 2.1 and 2.2 using the position of 1.1 and 1.2 as references.

**Figure 13:**
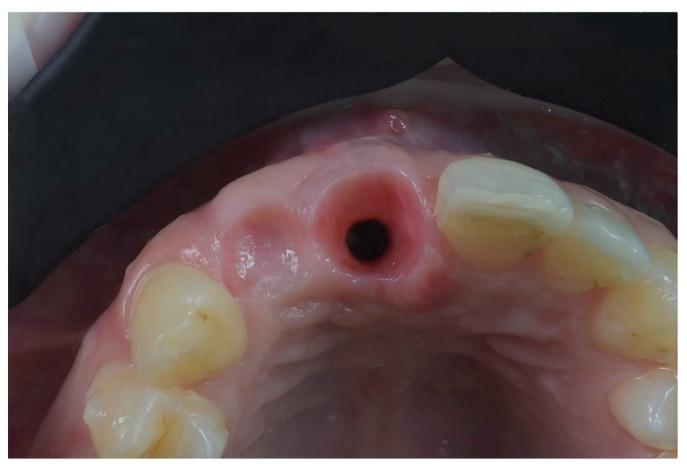
Case 2. Dynamic compression of the soft tissues.

**Figure 14:**
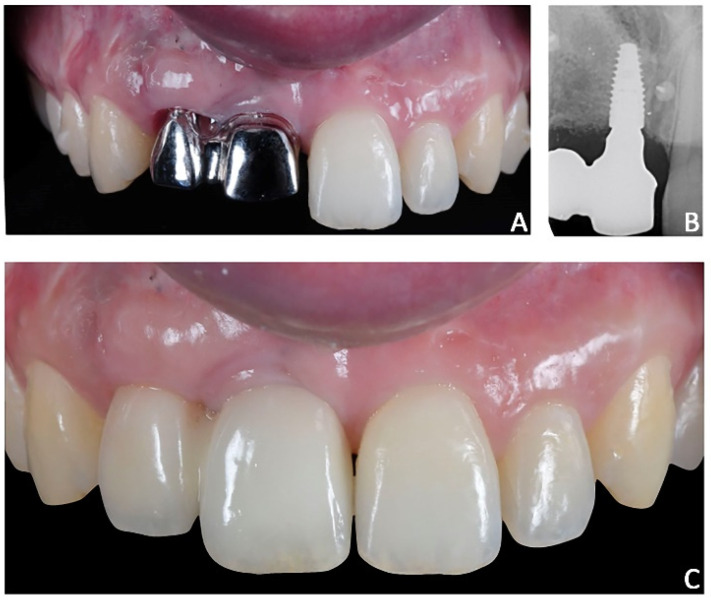
Case 2. Final prosthesis using milled cobalt-chrome structure and feldspathic porcelain. (**A**) Milled cobalt-chrome structure; (**B**) radiographic evaluation; (**C**) prosthesis veneered with feldspathic porcelain.

The first part of the therapy consisted of removing the causes of inflammation in the anterior segment, respectively, in the edentulous area removing the fixing screw and around natural teeth by performing professional oral hygiene and scaling (Figure 8B). After that, the treatment plan for implant-prosthetic rehabilitation of the 2.1 was performed and supported by digital systems:

1. Prosthetically guided implant planning. After removing the provisional prosthesis, an intra-oral scan was recorded, and the lab technician was asked to produce a virtual wax-up of the ideal position of the teeth. A Cone-Beam Computed Tomography (CBCT) was performed, and the 3D rendered model of the maxilla was registered with the intra-oral scan using the implant planning software Co-Diagnostix (Straumann, Montreal, Canada) (Figure 9). The software was used to plan the implant’s ideal position and design a surgery guide. Figure 10 shows the clinical procedure of the guided implant surgery. Once the osseointegration occurred, an intra-oral scan was performed to register the position of the implant and the surrounding soft tissues with the healing abutment in place (Figure 11A,B).

2. Positioning of the provisional prosthesis (11 with cantilever 1.2). The scan file was sent to the lab technician, which was asked to design a fixed temporary prosthesis in polymethyl methacrylate (PMMA), screwed onto the implant, and with the following instructions: (1) designing the 1.1 and 1.2 with the same size of contralateral teeth, and (2) maintaining the ideal overjet and overbite ratio compared to opposite arch (Figure 11C).

3. Orthodontic set-up. The orthodontic treatment aimed to optimize the position of 2.1 and 2.2 using clear aligners. In the Clincheck software, the linguo-vestibular inclination of the 2.1 and 2.2 were corrected using the position of the temporary elements (i.e., 11 and 12) as the anatomical limit for the orthodontic movement. The programmed orthodontic movement was staged, and seven aligners were required to achieve the final pre-prosthetic teeth position. The patient wore each aligner for 2 weeks for a total treatment time of 14 weeks (Figure 12). During the therapy with systematic Invisalign GO, dynamic compression of the soft tissues was performed in both the pontic region and the intra-mucosal region (Figure 13).

4. Final prosthetic rehabilitation. After orthodontic treatment, two intra-oral scans were registered: (1) an intra-oral scan with the position of the implant and surrounding soft tissues and (2) an intra-oral scan with the provisional prosthesis. The two files were sent to the laboratory and would replicate the relationships between the soft tissues and the temporary prosthesis in the final prosthesis. After prototyping the 3D model of the maxillary arch, the lab technician projected a milled cobalt-chrome structure that was veneered with feldspathic porcelain (Figure 14).

The rigorous planning of the prosthetic rehabilitation, including the orthodontic treatment, allowed us to reach an extraordinarily satisfactory and predictable result from aesthetic and functional perspectives.

### 1.3. Case 3

**Figure 15:**
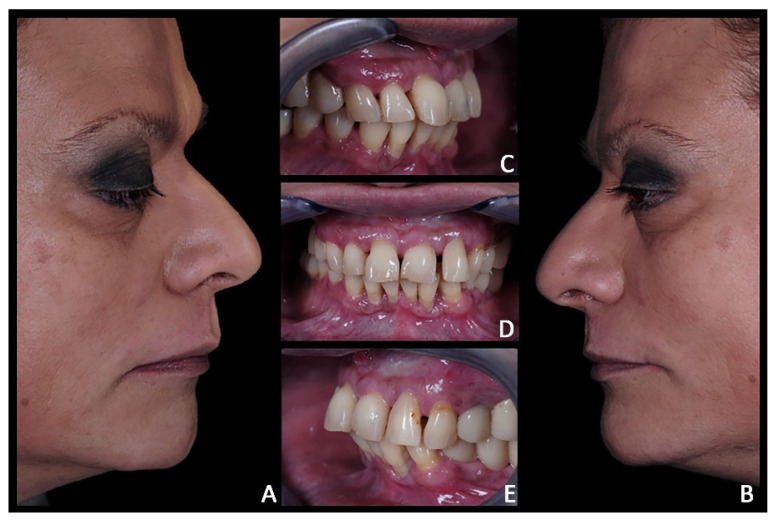
Case 3. Pre-treatment patient characteristics: extra-oral and intra-oral photographic records. (**A**) Extra-oral right profile view; (**B**) extra-oral left profile view; (**C**) intra-oral lateral right view; (**D**) intra-oral frontal view; (**E**) intra-oral lateral left.

**Figure 16:**
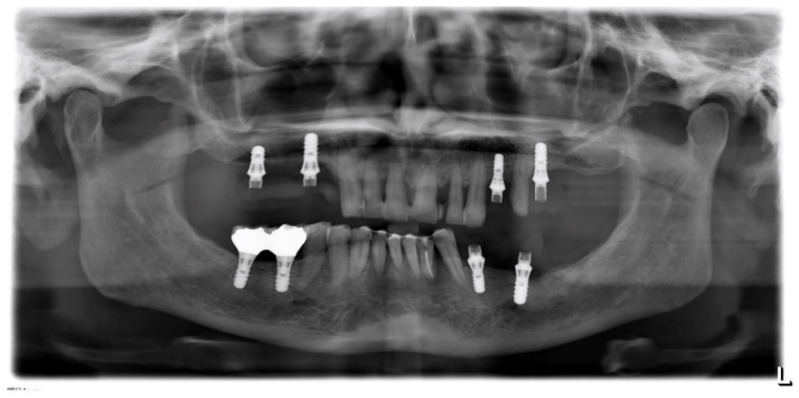
Case 3. Panorex.

A 59-year-old female attended a consultation complaining of aesthetic dissatisfaction with the diastemas between her upper frontal teeth and difficulty speaking and chewing. The patient wore provisional crowns supported by implants in the posterior regions (Figure 15). After an accurate diagnostic phase, which involved (1) intra-oral radiographic status, (2) probing of natural elements and implants, and (3) mobility and occlusal evaluation, the patient was classified as affected by periodontitis of stage 4 (Figure 16). Strong occlusal contacts were detected between upper and lower incisors, suggesting that occlusal trauma could contribute to the severity of the incisors’ mobility. In this regard, the provisional posterior crowns were maintaining the previous (reduced) vertical dimension without mitigating anterior contacts.

**Figure 17:**
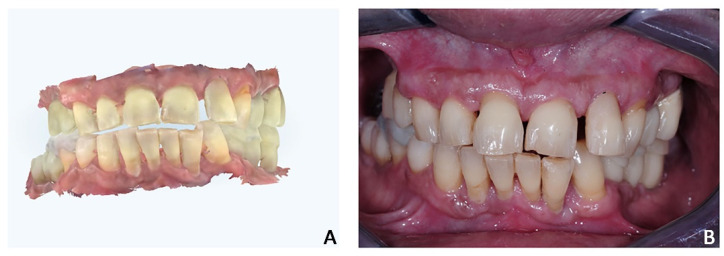
Case 3. Digital design of posterior bites (see the increment of vertical dimension and the anterior open-bite). (**A**) Intra-oral scans; (**B**) intra-oral photograph with cemented posterior multiple bites.

**Figure 18:**
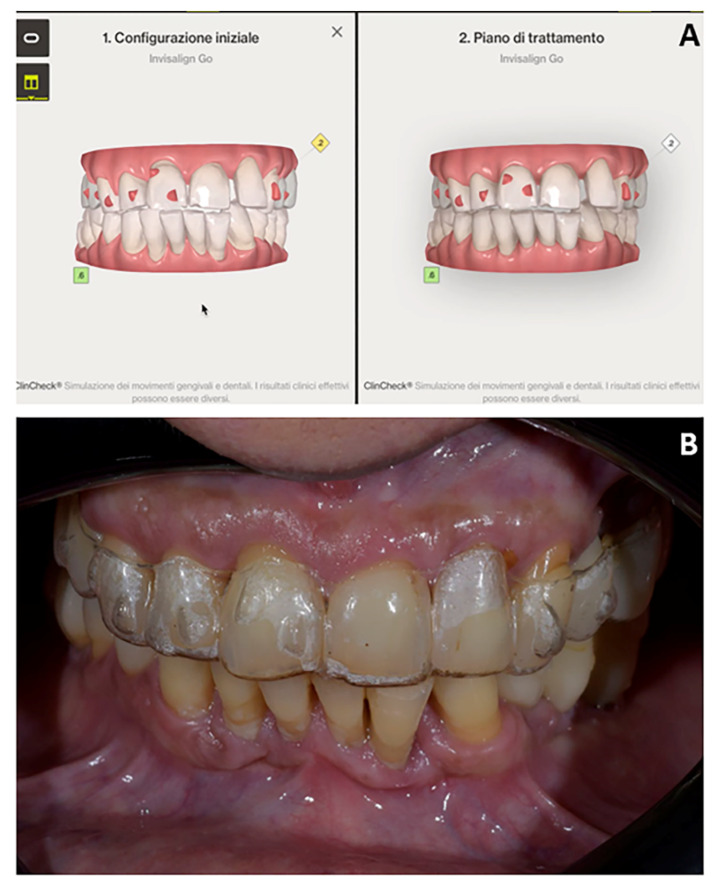
Case 3. (**A**) Orthodontic set-up (Invisalign Go platform); (**B**) intra-oral photograph with clear aligner.

**Figure 19:**
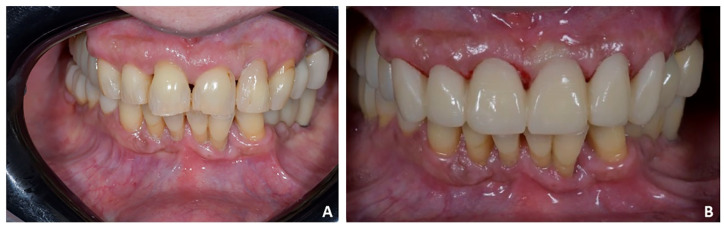
Case 3. (**A**) Posterior temporary crowns were replaced with monolithic zirconia crowns screwed on implants; (**B**) provisional crowns in the upper anterior segment from 1.3 to 23.

**Figure 20:**
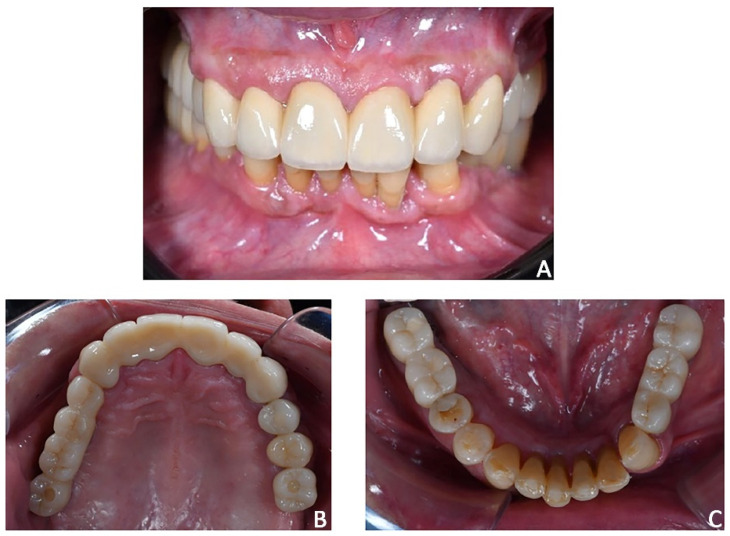
Case 3. The case was finalized by replicating the information functional characteristics of the provisional crowns in the final definitive prosthesis. (**A**) Intra-oral frontal view; (**B**) occlusal upper view; (**C**) occlusal lower view.

The digital diagnostic workflow, treatment strategy, and execution were performed as follows:

1. Initial therapy. The patient underwent respectively surgical and non-surgical periodontal therapy. The combination of both procedures allowed for the restoration of the physiology of the periodontal state, as confirmed by a probing depth that was contained within 4 mm.
2. Prosthetic evaluations. The patient showed a reduced posterior vertical dimension that caused a slight forward shift of the mandible with the generation of strong anterior contacts between the upper and lower incisors. This contributed to the mobility of the frontal teeth and the increased flaring and appearance of diastema.
3. Prosthetic treatment plan. Intra-oral scans were acquired, and a new vertical dimension was registered. In particular, the digital inter-arches relationship was recorded with 3 mm of free space in the posterior region. The digital vertical dimension recorded served as a reference to design multi-crown posterior provisional bites digitally. Afterward, the bites were 3D printed and cemented over the lower provisional crowns. This strategy allowed for an increase in the Occlusal Vertical Dimension (OVD) by re-establishing facial vertical aesthetic proportions and reducing the secondary occlusal trauma between upper frontal elements (Figure 17A,B).

4. Orthodontic set-up. The orthodontic treatment aimed to optimize the position of the anterior teeth for the final prosthetic rehabilitation. In particular, the treatment plan involved the reduction of incisors flaring and diastemas to correct incisal guidance and better support prosthetic crowns and smile aesthetics. The programmed orthodontic movement was staged, and eight aligners were required to achieve the final pre-prosthetic teeth position. The patient wore each aligner for two weeks for a total treatment time of 4 months (Figure 18A,B).

5. Prosthetic finalization. After orthodontic treatment, posterior rehabilitation was completed by replacing the temporary crowns with monolithic zirconia crowns screwed on implants (Figure 19A). In the same appointment, provisional crowns were applied in the upper anterior segment from 1.3 to 23 and kept for one month (Figure 19B). After the final aesthetic and functional analysis wards, the case was finalized by replicating the information and functional characteristics of the provisional crowns in the final definitive prosthesis (Figure 20). The upper fixed prosthesis was zirconia-ceramic, stratified in the vestibular (not functional) area.

**Figure 21:**
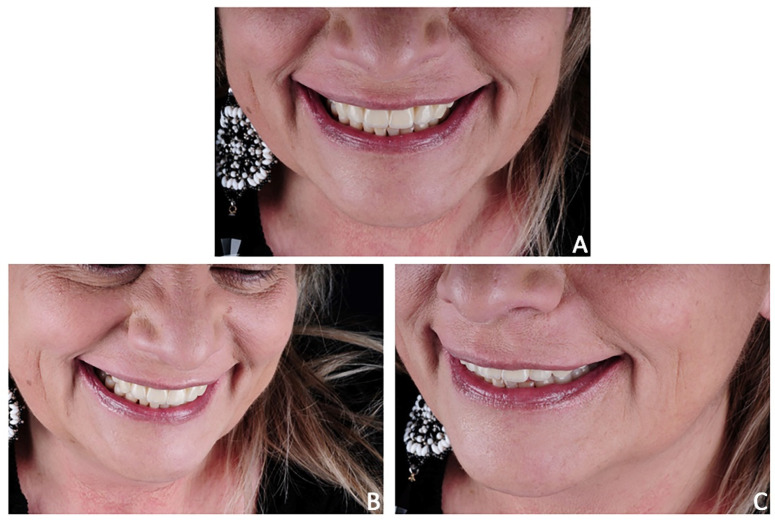
Case 3. Post-treatment smile aesthetics. (**A**) frontal smile view; (**B**) 3/4 right smile view; (**C**) 3/4 left smile view.

The planning of the prosthetic rehabilitation, including the augmentation of vertical dimension and the orthodontic treatment, allowed for the achievement of an extremely satisfactory and predictable result from aesthetic and functional perspectives (Figure 21).

## 2. Discussion

Conformative rehabilitations represent a significant part of daily dental practice due to the increased demand for aesthetic treatments from adult patients. Over the last decades, patients’ behavior related to oral health prevention has significantly improved. Thus, patients who refer to dental offices generally feature less edentulism than in the past and are more interested in aesthetic solutions rather than improving function. In addition, patients’ aesthetic expectations have tremendously grown, probably in conjunction with the advent of social media and people’s desire to share and “immortalize” daily moments with photos, selfies, or videos [24,25,26]. In this scenario, the role of the clinicians is to find the ideal compromise between the patient’s aesthetic expectations and the functional outcomes.

The cases discussed hereby reflect this approach, and thanks to digital instruments and pre-prosthetic orthodontics, it has been possible to achieve predictable results with the maximum satisfaction of both patients and the doctor. Patients were satisfied since they reached their aesthetic expectations in a short period, while the doctor was satisfied since he accomplished the aesthetic rehabilitations without negatively interfering with function and respecting the biological principles of minimally invasive preparations.

Orthodontic movements have been programmed according to the final prosthetic rehabilitations, following the principle of prosthetic-guided orthodontics (PGO). In this regard, PGO finds more and more applications in modern dental treatments, most of which need to be multidisciplinary to be predictable and biologically oriented. Modern dentistry aims to rehabilitate patients in terms of function, aesthetics, and phonetics while trying to be minimally invasive. The necessity of orthodontic tooth movement prior to prosthetic rehabilitation is fundamental for some patients to optimize both aesthetic and functional aspects of dental treatment. However, this topic has not been supported by adequate scientific evidence since most articles regarding pre-prosthetic orthodontics, including the present report, are clinical reports supported by clinicians’ opinions and without comparative assessment [27]. However, these reports can provide useful information to dentists in recognizing which patients could benefit from orthodontics intervention and understanding how orthodontic treatment can be utilized to improve the prognosis of prosthetic rehabilitation.

Generally, the treatment plan for patients requiring pre-prosthetic orthodontics must always begin with a diagnostic wax-up. This important tool enables both the orthodontist and the prosthodontist to visualize the final results. The orthodontist should confirm that the virtual changes in tooth position can be clinically achievable; the prosthodontist should confirm that the planned tooth position is ideal for the future prosthesis, considering both aesthetic and functional parameters. In this regard, the Invisalign GO system has simplified the treatment plan stages of the PGO approach since the digital pre-visualization of the outcomes represents a “common field” where prosthodontists and orthodontists can interact and make the best decision according to patients’ needs. The final occlusion can be customized according to the individual’s dental arch form and preferences for smile aesthetics. Adequate communication between prosthodontists (or restorative dentists), orthodontists, and other dental specialists is crucial to achieving programmed clinical outcomes; each specialist should know when to intervene, the treatment time required to reach the established objectives, and the cost of the provided treatment. At the same time, the patient expects to know this information before agreeing to the proposed treatment plan [28,29].

Furthermore, CAT has represented a “game changer” in the clinical management of multidisciplinary orthodontic treatment. Traditionally, orthodontists are trained to be reactive, which means that each biomechanical decision is made “reactively” based on the clinical response to the previous appliance’s activation [30]. The clear aligner technique requires a more “proactive,” disciplined approach. The final treatment can be previewed and becomes an integral part of the complex prosthetic rehabilitation treatment. This requires a paradigm shift in the thought process from a “reactive” approach to a “proactive” approach. Another advantage of CAT is the possibility to customize the biomechanics by staging tooth movements in a specific sequence in the software program. The rate of tooth movement may also be adjusted according to the individual’s bone physiology by altering the scheduled number of days for aligner changes, depending on the individual’s response to tooth movement. This aspect is extremely important in adult patients affected by periodontal diseases or impacted teeth, where it is necessary to modulate the orthodontic movement from qualitative and quantitative perspectives [30,31,32].

However, it must be underlined that Invisalign Go is designed for minor orthodontic tooth movements and does not deal with the planning of complex orthodontic biomechanics or orthodontic treatment involving significant occlusal changes and requiring the biomechanical skills of experienced orthodontists. Since the authors’ considerations are based on clinical cases, future studies involving CAT systems for pre-prosthetic orthodontics are warmly encouraged.

## 3. Conclusions

Aesthetic rehabilitations require a multidisciplinary approach to satisfy patients’ expectations and respect the orofacial biological and functional principles. Prosthetically guided orthodontics (PGO) optimizes the effectiveness of aesthetic rehabilitation and the maintenance of biological parameters of tooth preparation. At the same time, the Invisalign GO system provides clinicians with a comprehensive digital platform to plan pre-prosthetic orthodontic movement and generate an aesthetic orthodontic solution with clear aligners.

## Data Availability

Data are available from the corresponding author upon reasonable request.
